# Implementation of a MS/MS database for isoquinoline alkaloids and other annonaceous metabolites

**DOI:** 10.1038/s41597-022-01345-y

**Published:** 2022-06-06

**Authors:** Salemon Akpa Agnès, Timothée Okpekon, Yvette Affoué Kouadio, Adrien Jagora, Dimitri Bréard, Emmanoel V. Costa, Felipe M. A. da Silva, Hector H. F. Koolen, Anne-Marie Le Ray-Richomme, Pascal Richomme, Pierre Champy, Mehdi A. Beniddir, Pierre Le Pogam

**Affiliations:** 1 Équipe “Chimie des Substances Naturelles” Université Paris-Saclay, CNRS, BioCIS, 5 rue J.-B. Clément, 92290 Châtenay-Malabry, France; 2 Laboratoire de Constitution et Réaction de la Matière (LCRM), UFR Sciences des Structures de la Matière et Technologie, Université Félix Houphouët-Boigny, BP 582 Abidjan 22, Côte d’Ivoire; 3 Univ Angers, SONAS, SFR QUASAV, F-49000 Angers, France; 4 Departamento de Química, Universidade Federal do Amazonas, Av. Rodrigo Otávio 1200, 69067-005 Manaus, AM Brazil; 5 Centro de Apoio Multidisciplinar (CAM), Universidade Federal do Amazonas, Av. Rodrigo Otávio 1200, 69067-005 Manaus, Brazil; 6 Grupo de Pesquisa em Metabolômica e Espectrometria de Massas, Universidade do Estado do Amazonas, Av. Carvalho Leal 1777, 69065-001 Manaus, Brazil

**Keywords:** Metabolomics, Mass spectrometry

## Abstract

This data descriptor reports on the upload to a public repository (GNPS) of the IQAMDB, IsoQuinoline and Annonaceous Metabolites Data Base, comprising 320 tandem mass spectra. This project originated from our in-house collection of isoquinolines. The diversity of compounds included in this database was further extended through the contribution of two additional laboratories involved in isoquinoline alkaloids research: University of Angers and University of Manaus. The generated MS/MS data were processed and annotated on an individual basis to promote their straightforward reuse by natural product chemists interested in either the description of new isoquinoline alkaloids or the dereplication of isoquinoline-containing samples. The interest of the current repertoire for dereplication purposes has been validated based on the molecular networking of the well-investigated plant model *Annona montana* against the IQAMDB‐implemented GNPS.

## Abstract (summary)

**Table:** 

Measurement(s)	electrospray ionization
Technology Type(s)	Ultra High-performance Liquid Chromatography • Tandem Mass Spectrometry

## Background & Summary

As one of the largest groups of plant alkaloids, isoquinolines include a significant number of well-known drugs and lead-compounds, such as morphine, codeine, noscapine, papaverine, D-tubocurarine, berberine, emetine or higenamine. These illustrious leads tantalized many laboratories to explore the phytochemistry of isoquinoline-producing plants, so that more than 3000 BenzylIsoQuinolines (BIQ) alkaloids are currently catalogued^1^, displaying varied and often significant bioactivities^2^. From a phylogenetic standpoint, (tetrahydro)benzylisoquinoline alkaloids are reported to occur in more than 40 different families with the most represented producers being Amaryllidaceae, Annonaceae, Berberidaceae, Fumariaceae, Hernandiaceae, Lauraceae, Menispermaceae, and Papaveraceae^3^. In the quest for alternative industrial processes of natural product supplies, a few isoquinoline alkaloids of outstanding interest succumbed to biotechnological manufacturing, such as morphine^4^ (and codeine as its biosynthetic intermediate) or noscapine^5^. The very last years witnessed tremendous advances in the metabolic engineering of BIQ with the development of optimized yeast strains synthesizing a considerable amount of (*S*)-reticuline, a biosynthetic pillar in the TetraHydroIsoQuinoline (THIQ) series, but also a diversity of unprecedented BIQ derivatives^1^. These advances in THIQ metabolite production guided Courdavault *et al*. to identify this family of compounds as holding “an eminent biosynthetic potential in the field of drug discovery”^6^.

Our continuous efforts towards the improvement of the molecular-networking pipeline efficiency through the upload of MS/MS data related with collections of structurally homogeneous natural products led us to implement two different databases today incorporated into the Global Natural Products Social Molecular Networking (GNPS) libraries^7^. A first contribution to these public repositories was dedicated to Monoterpene Indole Alkaloids^8^ (so-called MIADB), implying an international consortium of eight different natural product laboratories that resulted in a collection of 172 initial entries, now reaching more than 220 compounds. Our second initiative in the field, the LDB (Lichen Data Base), garnered 241 structurally diverse lichen phenolic compounds based on a collaborative project with University of Rennes 1 and the Berlin Garden and Botanical Museum^9^. We foresaw the possibility of building and deploying a third MS/MS database on the basis of the large diversity of isoquinoline alkaloids that have been isolated in Paris-Saclay^10^.

Described by Jussieu in 1789, the Annonaceae family comprises more than 120 genera and about 2100 species, all occurring in tropical and subtropical regions^11^. From a phytochemical standpoint, the annonaceous species have been vastly investigated, with a pronounced emphasis on their alkaloidal components^12^. Heretofore, more than 800 alkaloids have been reported from Annonaceous source^12^. Annonaceous alkaloids are vastly dominated by isoquinolines, mostly falling into benzyl- and bisbenzyltetrahydroisoquinolines, protoberberines, tetrahydroprotoberberines, proaporphines, aporphines, 7-substituted aporphines, oxoaporphines and phenanthrenes, all deriving from benzylisoquinoline precursors. A few non-isoquinoline alkaloids extend the diversity of Annonaceous metabolites through unusual structures, such as canangine, a napthyridine alkaloid^13^, original pyrimidine β-carboline structures referred to as annomontines^14,15^, a few prenylindoles and some indolosesquiterpenes^16^. It seems that the discovery rate of new chemical entities from Annonaceous sources decreases across decades as most of the recently published investigations in the field report known metabolites^17,18^. Although now uncommon, some original structures are still being unearthed from these plants, such as a few aristolactam alkaloids recently obtained from the thailandese *Dasymaschalon dasymaschalum* (Blume) I.M. Turner^19^ and new isoquinolines, including an unprecedented 8-oxohomoaporphines from the Amazonian *Duguetia surinamensis* R. E. Fries^20^. It is tempting to hypothesize that the quest for new structures from these plants is made difficult by the occurrence of important amounts of recurrent structures in Annonaceous plants that are hard to obviate in the course of untargeted phytochemical investigations. In such context, molecular networking strategies have proved useful to select natural material deserving deeper chemical studies as well as to streamline the isolation of compounds of interest through a rational, hypothesis-driven workflow^21^. This provides new opportunities to focus on minor, interesting compounds from deeply-dug plant material^22,23^. Nevertheless, the success of this dereplicative strategy primarily depends on the availability of reference tandem mass spectra uploaded to the GNPS spectral libraries^7^. Regarding the specific example of Annonaceous plants, some molecular networking-based investigations undertaken by some of us, involving an in-house collection of alkaloid standards, efficiently assisted the targeting of new compounds for purification and subsequent structure elucidation^20^. These works gratifyingly outlined the trend of Annonaceous alkaloids to clusterize in a scaffold-dependent manner. Some fragmentation patterns were even proposed by the same authors^24,25^, leading to extend the annotation provided by this dereplication to untagged nodes based on the cursory examination of MS/MS spectra^20^.

**Fig. 1:**
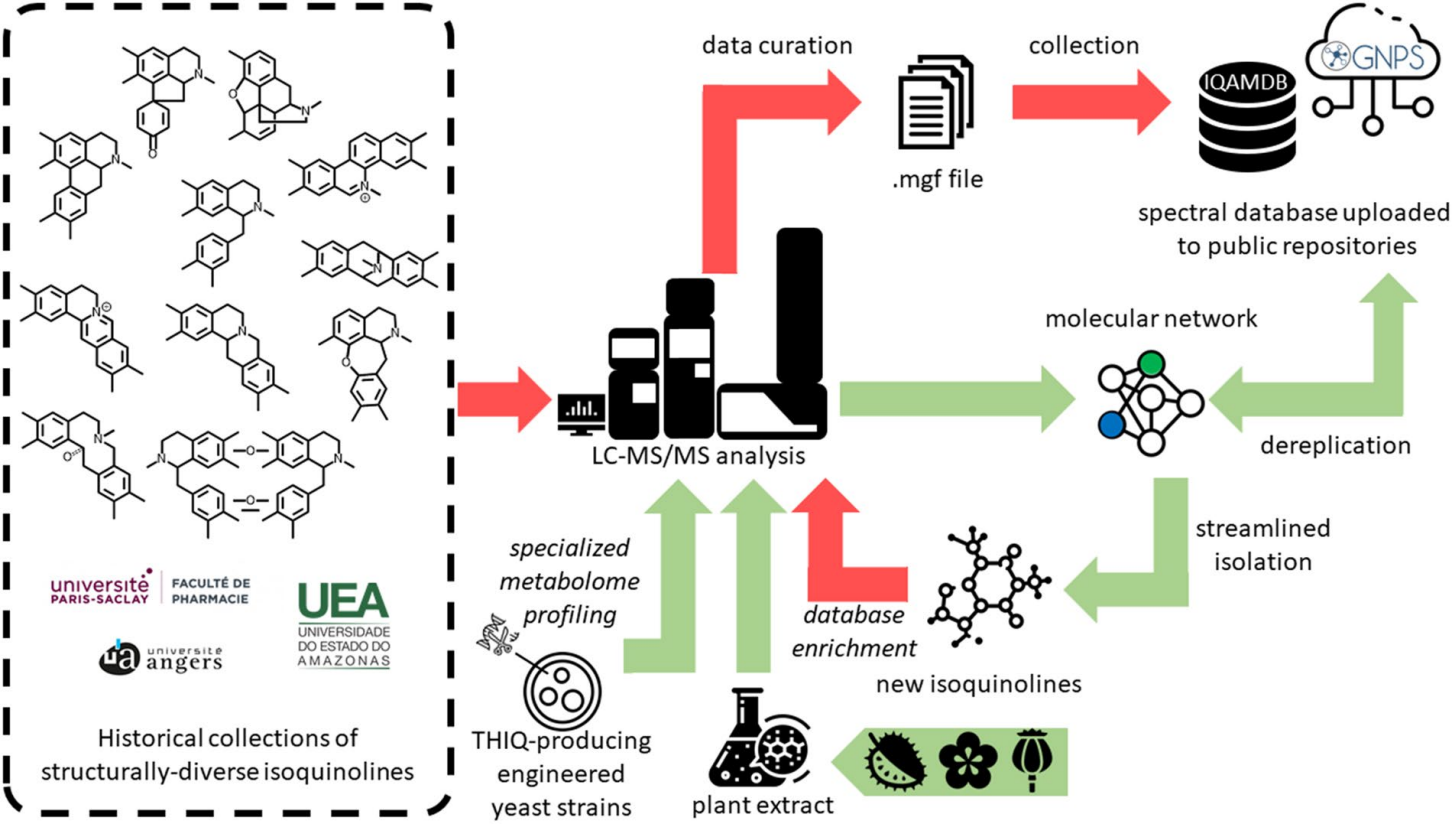
IQAMDB implementation workflow (red path) and pipeline for its subsequent use in the frame of a molecular networking-based dereplication strategy (green arrows).

We felt that our in-house collection of isoquinolines could set the ground for building a MS/MS database dedicated to Annonaceous metabolites. The core constitution of this Annonaceous and isoquinoline database was enlarged by inviting prominent natural products researchers in the field to share their collections with us. This collaborative work took advantage of additional Annonaceous isoquinolines included in the collections of Federal University of Amazonas and Amazonas State University (Manaus, Brazil). To broaden the applicability of our database, this combined collection was further extended to structurally-related isoquinolines from non-Annonaceous plant sources through the incorporation of compounds obtained from University of Angers (France) which comprised isolates from Menispermaceous, Hernandiaceous, Lauraceous and Papaveraceous species^26–28^. These contributions led us to constitute the largest MS/MS dataset of isoquinolines to date, namely the IQAMDB (IsoQuinolines and Annonaceous Metabolites DataBase) (https://gnps.ucsd.edu/ProteoSAFe/gnpslibrary.jsp?library = IQAMDB) (Fig. 1 and Table S1)^29^. This database should help pinpointing new chemical entities from isoquinoline-producing plants. We also hope that the upload of this database will be of interest to increase the reliability of the dereplication workflow in the frame of the very dynamic metabolic engineering efforts currently devoted to THIQ alkaloids. This data descriptor reports on the deposition of IQAMDB in the GNPS libraries and on its subsequent technical validation.

## Methods

### Sample preparation

The different standard samples were dissolved in HPLC-MS grade methanol at 0.5 mg/mL and placed into 1500 μL vials for analysis. For the alkaloidic extract, 1 g of dried and milled *Annona montana* Macfad. root bark was extracted using 50 mL of 0.25 M H_2_SO_4_ for one hour. The phase was alkalinized to a pH of 12 and counter-extracted three times using 35 mL of methylene chloride. The combined organic phases were evaporated in vacuo and dissolved in analytical grade methanol at a concentration of 0.5 mg/mL for UPLC/HRMS² analysis. Solvents were purchased from Sigma-Aldrich.

### Data acquisition

Samples were analyzed using an Agilent 6546 Accurate-Mass Q-TOF hyphenated with a 1290 Agilent Infinity II LC system. The chromatographic system was fitted with a Zorbax RRHD Eclipse Plus C18 column (2.1 × 50 mm, 1.8 μm). Elution solvents used were Milli-Q water + 0.1% formic acid (A) and acetonitrile + 0.1% formic acid (B), eluted using the following gradient: 5% B at 0 min; 100% B at 8 min, linear gradient; 8–12 min 100% B; 12–16 min 5% B. The flow rate was 500 µL.min^−1^. Mass spectrometry settings were as follows: capillary temperature at 320 °C, source voltage at 3.5 kV, and a sheath gas flow rate at 10 L/min. The mass spectrometer operated in positive polarity. Mass spectrometric acquisitions were divided into four scan events: positive MS with a window from *m/z* 100 to 1200, followed by three data-dependent MS/MS scans of the three most intense ions detected through the first scan event. Tandem mass spectrometric parameters were set as follows: collision energy = 50 eV, default charge of 1, isolation width of *m/z* 1.3. Dynamic exclusion was disabled. Purine (C_5_H_4_N_4_, *m/z* 121.050873), and HP-0921 (hexakis(*1H*, *1H*, *3H*-tetrafluoropropoxy)-phosphazene C_18_H_18_F_24_N_3_O_6_P_3_, *m/z* 922.009798 were used as internal lock masses. Full scans were acquired at a resolution of 60.000 (*m/z* 922) and 35.000 (*m/z* 121).

### Database constitution

The analysis of all these substances resulted in 320 files with the standard Agilent.d format. The list of features detected in every sample was generated following the auto MS/MS data mining process implemented in MassHunter software on every single file. Within the list of detected features, the exact mass of the feature of interest was identified and the other features were filtered out. The MS/MS data related to the signal of interest were subsequently converted into a .mgf file using a tailored intensity threshold thanks to the dedicated “Export” option of the MassHunter software.

### Molecular networking parameters (using IQAMDB standards as an input)

Every single MS/MS spectrum related to the IQAMDB standards was curated as indicated in the Database constitution section. A molecular network was then created using all the .mgf files as an input, using the online molecular networking workflow (version release_28.2) at GNPS^7^ (http://gnps.ucsd.edu) with a parent mass tolerance of 0.02 Da and a MS/MS fragment ion tolerance of 0.02 Da. The data were not clustered with MS‐Cluster. A network was then created where edges were filtered to have a cosine score above 0.6 and more than 6 matched peaks. Further edges between two nodes were kept in the network if and only if each of the nodes appeared in each other’s respective top 10 most similar nodes. The spectra in the network were then searched against GNPS spectral libraries. All matches kept between network spectra and library spectra were required to have a score above 0.6 and at least 6 matched peaks. The molecular networking data were analyzed and visualized using Cytoscape (ver. 3.6.0)^30^.

### Feature-based molecular networking parameters (dereplication of *A. montana*)

The MS2 data files related to the alkaloidic extract of *Annona montana* were converted from the .d (Agilent) standard data-format to .mzXML format using the MSConvert software, part of the ProteoWizard package^31^. The .mzXML file was further processed using MZmine 2 v53^32^. Mass detections were performed using a noise level threshold at 2E3 in MS1 and at 1.5E1 in MS2. The ADAP chromatogram builder used a minimum group size of scans of 2,a group intensity threshold of 2E3, a minimum highest intensity of 2E3 and a *m/z* tolerance of 10 ppm^33^. The chromatogram deconvolution used the Local Minimum Search algorithm with the following settings: chromatographic threshold = 1%, search minimum in RT range (min) = 0.05, minimum relative height = 2%, minimum absolute height = 1E3, min ratio of peak top/edge = 0.9, peak duration range (min) = 0.00–1. MS2 scans were paired using a *m/z* tolerance range of 0.02 Da and RT tolerance range of 0.15 min. Isotopes were grouped using the isotopic peaks grouper algorithm with a *m/z* tolerance of 10 ppm and a RT tolerance of 0.5 min keeping the most intense peak as the representative isotope. The peak list was filtered to keep only rows with MS2 features. The .mgf and .csv files were exported using the MZmine2 built-in “Export/Submit to GNPS /FBMN” option. The molecular network was finally created using the online FBMN workflow (version release_28.2) at GNPS (http://gnps.ucsd.edu) with a parent mass tolerance of 0.02 Da and a MS/MS fragment ion tolerance of 0.02 Da, where edges were filtered to have a cosine score above 0.6 and more than 6 matched peaks. Further edges between two nodes were maintained only if each of the nodes appeared in each other’s respective top 10 most similar nodes. The spectra in the network were then searched against GNPS spectral libraries. Matches between network spectra and library spectra were required to have a cosine score above 0.6 and at least 6 matched peaks. The molecular networking data were analyzed and visualized using Cytoscape (ver. 3.6.0)^30^. The obtained molecular network can be accessed at: https://gnps.ucsd.edu/ProteoSAFe/status.jsp?task = 3ff5f1048c7948ff96627438ac905acd.

## Data Records

Data reported in this article have been uploaded to the GNPS platform. Each MS² spectrum of the 320 compounds is assigned an individual accession number on the GNPS. The .mgf files were deposited and are publicly available at the MassIVE repository (MSV000088909) (10.25345/C55D8NG3T)^34^. The spectral collection is available for download from the library webpage of the GNPS (https://gnps.ucsd.edu/ProteoSAFe/libraries.jsp)^29^.

## Technical Validation

### Spectroscopic validation of IQAMDB compounds

The structure elucidation of the alkaloids implemented in the IQAMDB relied on an extensive set of spectroscopic techniques comprising at least nuclear magnetic resonance spectroscopy and high-resolution mass spectrometry. Further analyses were carried out whenever needed to ensure unambiguous structure assignment. These analyses were performed in the laboratory where the product had been isolated. The manual curation of each mass spectrometric file revealed the expected elemental composition, confirming that the samples had not degraded since they were isolated.

### Retained strategies for IQAMDB validation

The validation of the IQAMDB repertoire was achieved following two distinct strategies. At first, the topology of the molecular network obtained using the IQAMDB as an input was inspected to assess whether the uploaded MS/MS data could outline structural similarities between the standards included in the database, as a first indicator of the quality of IQAMDB spectrometric data. Then, the dereplication efficiency of the IQAMDB-implemented GNPS libraries was estimated by annotating the molecular network obtained from the alkaloidic extract of the thoroughly studied *Annona montana* Macfad.

### Topology of the molecular network obtained using the IQAMDB as an input

**Fig. 2:**
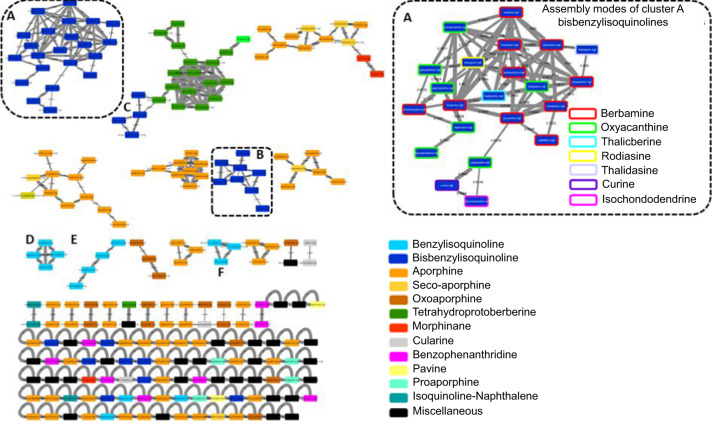
Scaffold-annotated molecular network using the MS/MS spectra of the IQAMDB as an input with a cosine similarity score cut-off of 0.6.

The molecular network generated using the tandem mass spectra of the IQAMDB as an input is disclosed in Fig. 2, with its nodes being colored according to their phytochemical class. Generally speaking, the topology of the molecular network does not deserve to be too thoroughly studied as it vastly depends on a wealth of different parameters, ensuring a low degree of reproducibility (mass spectrometric acquisition settings, molecular networking parameters, and even the diversity of compounds included in a molecular network that could instigate edges between some nodes or not). While having in mind these limits, we nevertheless felt relevant to outline a few clustering trends which may be of use to strengthen putative identifications against the IQAMDB-implemented GNPS. Chemical structures reported below to illustrate some rational clustering trends are provided in Figure S1 (Supporting Information).

The disclosed molecular network tended to cluster according to compounds structural class. The most striking examples of scaffold-based clustering are represented by tetrahydroprotoberberines (green nodes), benzylisoquinolines (light blue nodes) and bisbenzylisoquinolines (navy blue nodes)^35^. However, each of these scaffolds did not result in a unique and homogeneous cluster in the retained settings, sometimes conveying additional pieces of structural information. An example of rational structure-dependent clustering is that of bisbenzylisoquinolines which were split into three main clusters (A, B and C). As the largest group of isoquinoline alkaloids, bisbenzylisoquinolines are usually being classified based on their assembly modes, so that 26 different subtypes had been identified by Shamma and Moniot^36^. The benzylisoquinoline building blocks can indeed be joined by one or more ether bridges, sometimes accompanied by carbon-to-carbon biphenyl linkages or methyleneoxy junctions. Cluster A, exclusively, consisted of bisbenzylisoquinolines featuring two connections between their benzylisoquinoline components. Most nodes of this cluster corresponded to bisbenzylisoquinolines featuring a head-to-head ether connection and a tail-to-tail ether linkage between their monomeric units (berbamine, oxyacanthine, thalicberine and thalidasine subtypes). The rodiasine-type antioquine, featuring a head-to-head ether connection and a carbon-to-carbon biphenyl linkage as a tail-to-tail junction, connected with many representatives of the aforementioned subtypes. Notably, the southernmost part of cluster A comprised curine and isochondodendrine representatives, both featuring two head-to-tail ether connectivities. Cluster B connected bisbenzylisoquinolines featuring three intermonomeric connections. Most of the compounds fit the so-called trilobine-isotrilobine-micranthine type consisting of two ether bridges as head-to-head connections and an additional ether tail-to-tail linkage. The westernmost part of this cluster comprised tiliacorine and tiliacorinine disclosing two head-to-head ether connections and a biphenyl carbon-to-carbon bond tethering their tails. At last, four representatives loosely attached to the tetrahydroprotoberberine cluster (sub-cluster C). These molecules only featured one connection between their isoquinolines building blocks as a tail-to-tail junction (*viz*. daurisoline subtype). Likewise, the cursory examination of the clustering behaviour of other isoquinoline series revealed some tendencies which were seemingly related to some sharp structural features. Cluster D was composed of benzylisoquinolines substituted with a OH group on their benzyl component and by two oxygenated functionalities on their isoquinoline part. Benzylisoquinolines containing more than two methoxy groups on their isoquinoline component and a further methoxy functionality on their benzyl ring were detected in cluster E. At last, cluster F only collated structures disclosing a reticuline-type substitution pattern (a methoxy and a phenolic function on both isoquinoline and benzyl parts).

#### Validation of the IQAMDB against the phytochemically-defined *Annona montana* alkaloidic extract

The validation of the IQAMDB repertoire was based on the dereplication of *Annona montana*, commonly known as the mountain soursop, as a deeply-dug Annonaceous plant model. An alkaloidic extract was prepared from the historical sample of *A. montana* root bark, which had been phytochemically investigated by Lebœuf and co-workers in our laboratory in the 1980s^14,37^ and was further analyzed in positive polarity by UPLC-HRMS². To capture the chemical diversity of this extract, the UPLC−HRMS² data were processed using the feature-based molecular networking workflow^38^, and subsequently dereplicated against the IQAMDB, hosted by GNPS^7^. This pipeline resulted in dereplicating 27 unique and structurally-diverse compounds (Table 1). Notably, all these hits were exclusive to the IQAMDB, indicating the limited number of former Annonaceous compounds references in GNPS spectral libraries prior to the upload of the IQAMDB. Among those hits, 7 were previously reported from this plant source: Annomontine, methoxyannomontine^14^, argentinine^39^, atherosperminine^37^, coreximine^37^, liriodenine^39^, and reticuline^37^. Besides being closely related to some of these putative structures, 13 further hits had already been reported from other *Annona* species: benzyltetrahydroisoquinolines anomuricine and anomurine (both known from *A. muricata*^31^), *N*-methylcoclaurine (from *A. sericea*^40^), reticuline *N*‐oxide (*A. salzmanni*^41^) and tembetarine (tentatively known from *A. salzmanni*^41^); proaporphines such as glaziovine and stepharine, both reported from *A. purpurea*^42,43^; tetrahydroprotoberberines kikemanine (*A. glabra*^44^), pessoine (from *A. spinescens*^45^), and stepholidine (*A. cherimolia*^46^); aporphines such as nornuciferine (*A. muricata*^47^), obovanine (*A. coriacea*^48^) and roemerine (from both *A. squamosa*^49^ and *A. senegalensis*^50^). Most other hits corresponded to metabolites occurring in other Annonaceous genera. Among all the hits, only nandigerine was hitherto unknown from Annonaceous source. Even though this aporphine alkaloid is mainly related to Hernandiaceae^12^ and Lauraceae^51^, one of its derivatives, *N*-methylnandigerine *N*-oxide, had been reported from the Annonaceous *Polyalthia longifolia*^52^.

**Table 1:** Matches between the profiled compounds from an alkaloidic extract of *A. montana* root bark and IQAMDB.

Compound	Cosine	Comment
Annomontine	0.95	Described from *A. montana*^14^
Anomuricine	0.75	Described from other *Annona* species^53^
Anomurine	0.86	Described from other *Annona* species^53^
Argentinine	0.95	Described from *A. montana*^39^
Atherosperminine	0.97	Described from *A. montana*^37^
Atherosperminine-*N*-oxide	0.97	Known from other Annonaceous genera^17^
Bisnorargemonine	0.86	Known from other Annonaceous genera^54^
Coreximine	0.94	Described from *A. montana*^37^
Discoguattine	0.72	Known from other Annonaceous genera^17^
Glaziovine	0.64	Described from other *Annona* species^42^
Kikemanine	0.63	Described from other *Annona* species^44^
Liriodenine	0.96	Described from *A. montana*^37^
Methoxyannomontine	0.88	Described from *A. montana*^14^
*N-*methylcoclaurine	0.91	Described from other *Annona* species^40^
Nandigerine	0.72	Not known from Annonaceous source
Noratherosperminine	0.93	Known from other Annonaceous genera^55^
Norjuziphine	0.90	Known from other Annonaceous genera^56^
Nornuciferine	0.77	Described from other *Annona* species^47^
Obovanine	0.92	Described from other *Annona* species^48^
*O*-methylisopiline	0.64	Known from other Annonaceous genera^57^
Pessoine	0.80	Described from other *Annona* species^45^
Reticuline	0.88	Described from *A. montana*^37^
Reticuline *N*-oxide	0.88	Described from other *Annona* species^41^
Roemerine	0.95	Described from other *Annona* species^49,50^
Stepharine	0.85	Described from other *Annona* species^43^
Stepholidine	0.81	Described from other *Annona* species^46^
Tembetarine	0.74	Described from other *Annona* species^41^

#### Metadata

The MS/MS spectra of the IQAMDB library are associated to a variety of details including: LC-MS/MS acquisition parameters, RT (see Supporting Information), instrument details, smiles and InChi codes, structures, and chemical formula. These metadata are available on the GNPS website.

## Supplementary information

Chemical structures and metadata related to all the molecules included in the IQAMDB

## Data Availability

The LC-MS feature detection software (MassHunter®) used in this work is commercially available from Agilent®.

## References

[1] Pyne ME (2020). A yeast platform for high-level synthesis of tetrahydroisoquinoline alkaloids. Nature Commun..

[2] Nguyen VK, Kou KG (2021). The biology and total syntheses of bisbenzylisoquinoline alkaloids. Org. Biomol. Chem..

[3] Bisset, N. G. Plants as a source of isoquinoline alkaloids. in *The Chemistry and Biology of Isoquinoline Alkaloids* 1–22 (Springer, 1985).

[4] Galanie S, Thodey K, Trenchard IJ, Interrante MF, Smolke CD (2015). Complete biosynthesis of opioids in yeast. Science.

[5] Li Y (2018). Complete biosynthesis of noscapine and halogenated alkaloids in yeast. Proc. Natl Acad. Sci..

[6] Kulagina N, Papon N, Courdavault V (2021). Microbial Cell Factories for Tetrahydroisoquinoline Alkaloid Production. ChemBioChem.

[7] Wang M (2016). Sharing and community curation of mass spectrometry data with Global Natural Products Social Molecular Networking. Nat. Biotechnol..

[8] Ramos AEF (2019). Collected mass spectrometry data on monoterpene indole alkaloids from natural product chemistry research. Sci. Data.

[9] Olivier-Jimenez D (2019). A database of high-resolution MS/MS spectra for lichen metabolites. Sci. Data.

[10] Lebœuf M, Cavé A, Bhaumik PK, Mukherjee B, Mukherjee R (1980). The phytochemistry of the annonaceae. Phytochemistry.

[11] Hutchinson, J. The genera of flowering plants. Dicotylédones. Vol. 1. *The genera of flowering plants. Dicotylédones. Vol. 1*. (1964).

[12] Lúcio, A. S. S. C., de Almeida JR., da-Cunha, E. V. L., Tavares, J. F. & Barbosa Filho, J. M. Alkaloids of the Annonaceae: Occurrence and a Compilation of Their Biological Activities. in *The Alkaloids: Chemistry and Biology***vol. 74** 233–409 (Elsevier, 2015). 10.1016/bs.alkal.2014.09.002 25845063

[13] Lebœuf M, Cavé A (1976). Alkaloids of Annonaceae. XIV. Identification of canangine and eupolauridine, new alkaloids with a naphthyridine nucleus. Lloydia.

[14] Lebœuf, M. *et al*. Alkaloids of the Annonaceae. Part 33. Annomontine and methoxyannomontine, two new pyrimidine-β-carboline-type alkaloids from *Annona montana*. *J. Chem. Soc., Perkin Trans*. 1205–1208 (1982).

[15] Costa EV (2008). Full NMR analysis of annomontine, methoxy-annomontine and N-hydroxyannomontine pyrimidine-β-carboline alkaloids. Magn. Reson. Chem..

[16] Cave A, Guinaudeau H, Leboeuf M, Ramahatra A, Razafindrazaka J (1978). Alcaloïdes des Annonacées XVIII1: Alcaloïdes des Ecorces de tronc du *Polyalthia suaveolens* Engl. et Diels. Planta Med..

[17] de Souza Araújo M, da Silva FMA, Koolen HHF, Costa EV (2020). Isoquinoline-derived alkaloids from the bark of *Guatteria olivacea*(Annonaceae). Biochem. Syst. Ecol..

[18] Nardelli VB (2021). Isoquinoline-derived alkaloids and one terpene lactone from the leaves of *Duguetia pycnastera*(Annonaceae). Biochem. Syst. Ecol..

[19] Suthiphasilp V (2019). Dasymaschalolactams A–E, Aristolactams from a Twig Extract of *Dasymaschalon dasymaschalum*. J. Nat. Prod..

[20] Paz WH (2019). Structure-Based Molecular Networking for the Target Discovery of Oxahomoaporphine and 8-Oxohomoaporphine Alkaloids from *Duguetia surinamensis*. J. Nat. Prod..

[21] Fox Ramos AE, Evanno L, Poupon E, Champy P, Beniddir MA (2019). Natural products targeting strategies involving molecular networking: Different manners, one goal. Nat. Prod. Rep..

[22] Fouotsa H (2021). Voatriafricanines A and B, Trimeric Vobasine-Aspidosperma-Aspidosperma Alkaloids from *Voacanga africana*. J. Nat. Prod..

[23] Fouotsa H (2022). Pyrrovobasine, hybrid alkylated pyrraline monoterpene indole alkaloid pseudodimer discovered using a combination of mass spectral and NMR-based machine learning annotations. Org. Biomol. Chem..

[24] da Silva FM (2017). Positive electrospray ionization ion trap mass spectrometry and *ab initio* computational studies of the multi-pathway fragmentation of oxoaporphine alkaloids. Int. J. Mass Spectrom..

[25] Neto FC (2020). Characterization of aporphine alkaloids by electrospray ionization tandem mass spectrometry and density functional theory calculations. Rapid Commun. Mass Spectrom..

[26] Richomme P, Lavault M, Jacquemin H, Bruneton J (1984). Etude des Hernandiacées VI (1): lignanes et alcaloïdes de *Hernandia guianensis*. Planta Med..

[27] Chalandre M-C, Bruneton J, Cabalion P, Guinaudeau H (1986). Alcaloïdes de *Gyrocarpus americanus*. J. Nat. Prod..

[28] Lavault M (1987). Alcaloïdes bisbenzylisoquinoléiques de *Albertisia* cf. *A. papuana*. Can. J. Chem..

[29] *IsoQuinoline and Annonaceous Metabolites Data Base (IQAMDB)*, *GNPS*, https://gnps.ucsd.edu/ProteoSAFe/gnpslibrary.jsp?library=IQAMDB

[30] Shannon P (2003). Cytoscape: a software environment for integrated models of biomolecular interaction networks. Genome Res..

[31] Chambers MC (2012). A cross-platform toolkit for mass spectrometry and proteomics. Nature Biotechnol..

[32] Pluskal T, Castillo S, Villar-Briones A, Orešič M (2010). MZmine 2: modular framework for processing, visualizing, and analyzing mass spectrometry-based molecular profile data. BMC Bioinformatics.

[33] Myers OD, Sumner SJ, Li S, Barnes S, Du X (2017). One Step Forward for Reducing False Positive and False Negative Compound Identifications from Mass Spectrometry Metabolomics Data: New Algorithms for Constructing Extracted Ion Chromatograms and Detecting Chromatographic Peaks. Anal. Chem..

[34] Le Pogam P (2022). MassIVE.

[35] Shamma, M. The bisbenzylisoquinolines. in *The Isoquinoline Alkaloids. Chemistry and Pharmacology*. 115–152 (Academic Press, 1972).

[36] Shamma M, Moniot J (1976). The Systematic Classification of Bisbenzylisoquinolines. Heterocycles.

[37] Lebœuf M (1982). Alcaloides des annonacées XL: Etude chimique et pharmacologique des alcaloïdes de l’*Annona montana*. Plant. Medic. Phytother..

[38] Nothias L-F (2020). Feature-based molecular networking in the GNPS analysis environment. Nature Methods.

[39] Wu Y-C, Chang G-Y, Chang-Yih D, Shang-Kwei W (1993). Cytotoxic alkaloids of *Annona montana*. Phytochemistry.

[40] Campos FR (2008). Isoquinoline alkaloids from leaves of *Annona sericea* (Annonaceae). Biochem. Syst. Ecol..

[41] Lima JM, Leme GM, Costa EV, Cass QB (2021). LC-HRMS and acetylcholinesterase affinity assay as a workflow for profiling alkaloids in *Annona salzmannii* extract. J. Chromatogr. B.

[42] Chang F-R, Wei J-L, Teng C-M, Wu Y-C (1998). Antiplatelet Aggregation Constituents from *Annona purpurea*. J. Nat. Prod..

[43] Sonnet PE, Jacobson M (1971). Tumor inhibitors II: cytotoxic alkaloids from *Annona purpurea*. J. Pharm. Sci..

[44] Chang F-R, Chen C-Y, Hsieh T-J, Cho C-P, Wu Y-C (2000). Chemical Constituents from *Annona glabra* III. J. Chinese Chemical Soc..

[45] Queiroz EF, Roblot F, Cavé A, de Q. Paulo M, Fournet A (1996). Pessoine and spinosine, two catecholic berbines from *Annona spinescens*. J. Nat. Prod..

[46] Villar A, Mares M, Rios JL, Cortes D (1985). Alkaloids from *Annona cherimolia* leaves. J. Nat. Prod..

[47] Hasrat JA, De Bruyne T, De Backer J-P, Vauquelin G, Vlietinck AJ (1997). Isoquinoline derivatives isolated from the fruit of *Annona muricata* as 5-HTergic 5-HT(1A) receptor agonists in rats: Unexploited antidepressive (lad) products. J. Pharm. Pharmacol..

[48] Nogueira da Silva Avelino Oliveira Rocha G (2021). Chemical constituents from the leaves and branches of *Annona coriacea* Mart. (Annonaceae). Biochem. Syst. Ecol..

[49] Bhakuni DS, Tewari S, Dhar MM (1972). Aporphine alkaloids of *Annona squamosa*. Phytochemistry.

[50] You M (1995). (-)-Roemerine, an aporphine alkaloid from *Annona senegalensis* that reverses the multidrug-resistance phenotype with cultured cells. J. Nat. Prod..

[51] Teles MMRS (2019). Alkaloids of the Lauraceae. The Alkaloids: Chemistry and Biology.

[52] Wu YC (1989). Azafluorene and aporphine alkaloids from *Polyalthia longifolia*. Heterocycles.

[53] Lebœuf M (1981). Alcaloïdes des Annonacées XXIX: Alcaloïdes de l’*Annona muricata* L. Planta Med..

[54] Costa EV (2021). Benzylated Dihydroflavones and Isoquinoline-Derived Alkaloids from the Bark of *Diclinanona calycina* (Annonaceae) and Their Cytotoxicities. Molecules.

[55] Lu S-T, Wu Y-C, Leou S-P (1985). Alkaloids of formosan *Fissistigma* and *Goniothalamus* species. Phytochemistry.

[56] Chaves MH, De Santos LA, Lago JHG, Roque NF (2001). Alkaloids from *Porcelia macrocarpa*. J. Nat. Prod..

[57] Rasamizafy S, Hocquemiller R, Cavé A, Fournet A (1987). Alcaloïdes des Annonacées, 78. Alcaloïdes des Écorces d’un *Duguetia spixiana* de Bolivie. J. Nat. Prod..

