# Supplementary material for: Implementation of a MS/MS database for isoquinoline alkaloids and other annonaceous metabolites
Source: Sci Data. 2022 Jun 6;9:270. doi: 10.1038/s41597-022-01345-y (PMC9170680; doi:10.1038/s41597-022-01345-y)
Supplement: Supplementary file 1 — Chemical structures and metadata related to all the molecules included in the IQAMDB [file 41597_2022_1345_MOESM1_ESM.docx]

**Supplementary Information**

**Implementation of a MS/MS database for isoquinoline alkaloids and other annonaceous metabolites**

**Authors**

Salemon Akpa Agnès^1,2^, Timothée Okpekon^2^, Yvette Affoué Kouadio^1,2^, Adrien Jagora^1^, Dimitri Bréard^4^, Emmanoel V. Costa^5^, Felipe M. A. da Silva^6^, Hector H. F. Koolen^7^, Anne-Marie Le Ray-Richomme^4^, Pascal Richomme^4^, Pierre Champy^1^, Mehdi A. Beniddir^1^ & Pierre Le Pogam^1^

**Affiliations**

^1^Équipe “Chimie des Substances Naturelles” Université Paris-Saclay, CNRS, BioCIS, 5 rue J.-B. Clément, 92290 Châtenay-Malabry, France.

^2^Laboratoire de Constitution et Réaction de la Matière (LCRM), UFR Sciences des Structures de la Matière et Technologie, Université Félix Houphouët-Boigny, BP 582 Abidjan 22, Côte d’Ivoire.

^3^Département de Chimie Analytique, Minérale et Générale, Technologie Alimentaire, UFR Sciences Pharmaceutiques et Biologiques, Univ. FHB, 06 B. P. 2256, Abidjan 06, Côte d’Ivoire.

^4^Univ Angers, SONAS, SFR QUASAV, F-49000 Angers, France.

^5^Departamento de Química, Universidade Federal do Amazonas, Av. Rodrigo Otávio 1200, 69067-005 Manaus, Brazil Manaus, AM, Brazil.

^6^Centro de Apoio Multidisciplinar (CAM), Universidade Federal do Amazonas, Av. Rodrigo Otávio 1200, 69067-005 Manaus, AM, Brasil.

^7^Grupo de Pesquisa em Metabolômica e Espectrometria de Massas, Universidade do Estado do Amazonas, Av. Carvalho Leal 1777, 69065-001 Manaus, AM, Brasil.

Corresponding author: Pierre Le Pogam ([pierre.le-pogam-alluard@universite-paris-saclay.fr](mailto:pierre.le-pogam-alluard@universite-paris-saclay.fr)).

| Substance | Measured *m*/*z* | Source | Retention time (min) | Structure |
| --- | --- | --- | --- | --- |
| actinodaphnine | 312.1234 | Isolated | 2.08 |  |
| alpha-8-methylpseudoanibacanine | 312.1598 | Isolated | 1.83 |  |
| amurine | 326.1384 | Isolated | 2.33 |  |
| anaxagoreine | 284.1279 | Isolated | 1.69 |  |
| ancistrocladine | 408.2166 | Isolated | 2.74 |  |
| ancistrocladonine | 436.2477 | Isolated | 3.14 |  |
| ancistroealaensine | 422.232 | Isolated | 2.97 |  |
| anibacanine | 298.1441 | Isolated | 1.76 |  |
| annomontine | 262.1093 | Isolated | 2.90 |  |
| anolobine | 282.1122 | Isolated | 2.11 |  |
| anomuricine | 330.1708 | Isolated | 2.48 |  |
| anomurine | 344.1858 | Isolated | 2.77 |  |
| anonaine | 266.1175 | Isolated | 2.64 |  |
| antioquine | 609.2962 | Isolated | 2.00 |  |
| antofine | 364.1904 | Isolated | 2.82 |  |
| apateline | 549.237 | Isolated | 2.22 |  |
| apomorphine | 268.1332 | Synthesis | 1.68 |  |
| argemonine | 356.1851 | Isolated | 2.15 |  |
| argentinine | 296.1643 | Isolated | 2.71 |  |
| armepavine | 314.1750 | Isolated | 1.76 |  |
| asaronaldehyde | 197.0807 | Isolated | 2.81 |  |
| asiaticumine A | 284.084 | Isolated | 3.11 |  |
| asimilobine | 268.1329 | Isolated | 2.01 |  |
| atheroline | 338.1021 | Isolated | 2.41 |  |
| atherosperminine | 310.1810 | Isolated | 3.31 |  |
| atherosperminine *N*-oxide | 326.1752 | Isolated | 3.19 |  |
| beccapoline | 609.1657 | Isolated | 3.21 |  |
| berbamine | 609.2950 | Isolated | 2.07 |  |
| berberine | 336.1238 | Isolated | 2.80 |  |
| 10-beta-antofine-*N*-oxide | 362.1746  [M−H_2_O+H]^+^ | Isolated | 2.78 |  |
| bisnorargemonine | 328.1545 | Isolated | 1.88 |  |
| bisnoraromoline | 567.2488 | Isolated | 1.67 |  |
| boldine | 328.1550 | Isolated | 1.77 |  |
| caaverine | 268.1329 | Isolated | 2.24 |  |
| calafatine | 653.3215 | Isolated | 2.05 |  |
| calycinine | 312.1231 | Isolated | 2.05 |  |
| canangine | 205.0761 | Isolated | 2.96 |  |
| candicusine | 595.2805 | Isolated | 1.76 |  |
| celtine | 328.1547 | Isolated | 2.27 |  |
| cepharanthine | 607.2795 | Isolated | 2.31 |  |
| claviculine | 314.1393 | Isolated | 2.21 |  |
| cleistopholine | 224.0705 | Isolated | 2.88 |  |
| clolimalongine | 368.1258 | Isolated | 1.67 |  |
| coclaurine | 286.1436 | Isolated | 1.45 |  |
| coclobine | 607.2799 | Isolated | 2.35 |  |
| cocsoline | 549.2389 | Isolated | 2.14 |  |
| cocsuline | 563.2548 | Isolated | 2.10 |  |
| codeine | 300.1598 | Synthesis | 2.35 |  |
| colchicine | 400.1763 | Synthesis | 2.65 |  |
| colletine | 328.1914 | Isolated | 2.08 |  |
| columbamine | 338.1392 | Isolated | 2.37 |  |
| coreximine | 328.1552 | Isolated | 1.70 |  |
| corydaldine | 208.0966 | Isolated | 1.99 |  |
| corydine | 342.1698 | Isolated | 2.18 |  |
| corypalline | 194.1174 | Isolated | 1.22 |  |
| corypalmine | 342.1705 | Isolated | 2.29 |  |
| corytenchine | 342.1702 | Isolated | 1.87 |  |
| corytuberine | 328.1546 | Isolated | 1.61 |  |
| crebanine | 340.1539 | Isolated | 2.74 |  |
| crotsparine | 284.1287 | Isolated | 1.22 |  |
| cryptowoline | 326.1384 | Isolated | 1.74 |  |
| curicycleatjenine | 635.2747 | Isolated | 3.32 |  |
| curine | 595.2801 | Isolated | 2.03 |  |
| cycleanine | 623.3124 | Isolated | 2.15 |  |
| cycleatjehenine | 605.2643 | Isolated | 2.01 |  |
| daphnandrine | 595.2779 | Isolated | 2.03 |  |
| daphnoline | 581.2644 | Isolated | 1.68 |  |
| darienine | 272.0916 | Isolated | 2.69 |  |
| dauricine | 625.3267 | Isolated | 2.21 |  |
| daurisoline | 611.3103 | Isolated | 1.91 |  |
| 1,2-dehydroapateline | 547.2230 | Isolated | 2.02 |  |
| 1,2-dehydrotelobine | 561.2381 | Isolated | 2.46 |  |
| dehydrodiscretine | 338.1389 | Isolated | 2.35 |  |
| dehydronuciferine | 294.1497 | Isolated | 6.15 |  |
| dehydroroemerine | 278.1175 | Isolated | 6.35 |  |
| dehydrostephalagine | 308.1278 | Isolated | 6.58 |  |
| demethoxyguadiscine | 292.1332 | Isolated | 2.82 |  |
| 10-demethyldiscretine | 328.1542 | Isolated | 1.52 |  |
| 11-demethyldiscretine | 328.1544 | Isolated | 1.56 |  |
| 10-demethylxylopinine | 342.1698 | Isolated | 1.71 |  |
| dicentrinone | 336.0867 | Isolated | 2.56 |  |
| 6,6a-dihydrodemethoxyguadiscine | 294.1490 | Isolated | 2.88 |  |
| dihydroguatteriscine | 340.1906 | Isolated | 3.06 |  |
| dihydrosanguinarine | 334.1067 | Isolated | 6.10 |  |
| discoguattine | 326.1386 | Isolated | 2.29 |  |
| discretamine | 328.1547 | Isolated | 1.62 |  |
| discretine | 342.1703 | Isolated | 1.96 |  |
| domesticine | 326.1389 | Isolated | 2.20 |  |
| dragabine | 293.1281 | Isolated | 2.22 |  |
| duguetine *N*-oxide | 372.1446 | Isolated | 2.31 |  |
| duguevanine | 342.1337 | Isolated | 2.24 |  |
| elmerrillicine | 312.1230 | Isolated | 2.13 |  |
| emetine | 481.304 | Isolated | 5.12 |  |
| episteporphine | 296.1288 | Isolated | 2.23 |  |
| epi-ushinsunine | 296.1288 | Isolated | 2.53 |  |
| fagaronine | 350.14 | Isolated | 1.01 |  |
| fumariline | 352.116 | Isolated | 3.51 |  |
| glaucine | 356.1861 | Isolated | 2.33 |  |
| glaziovine | 298.1446 | Isolated | 1.22 |  |
| govadine | 328.1542 | Isolated | 1.51 |  |
| govanine | 342.1700 | Isolated | 2.51 |  |
| guacolidine | 340.1176 | Isolated | 2.02 |  |
| guacoline | 354.1343 | Isolated | 1.98 |  |
| guadiscidine | 308.1277 | Isolated | 2.45 |  |
| guadiscine | 322.1439 | Isolated | 2.95 |  |
| guattamine | 607.2803 | Isolated | 2.06 |  |
| guattaminone | 621.2592 | Isolated | 2.26 |  |
| guatterfriesidine | 336.1230 | Isolated | 2.72 |  |
| guatterine | 326.1389 | Isolated | 2.74 |  |
| guatterine-*N*-oxide | 342.1338 | Isolated | 2.73 |  |
| guatteriopsiscine | 356.1858 | Isolated | 2.55 |  |
| guattescidine | 310.1071 | Isolated | 1.88 |  |
| guattescine | 324.1231 | Isolated | 2.86 |  |
| habropetaline A | 394.2010 | Isolated | 2.47 |  |
| hernagine | 328.1547 | Isolated | 1.60 |  |
| hernovine | 314.1387 | Isolated | 1.23 |  |
| higenamine | 272.1285 | Isolated | 1.22 |  |
| hippadine | 264.066 | Isolated | 1.89 |  |
| homoaromoline | 609.2950 | Isolated | 1.92 |  |
| 14-hydroxyantofine | 380.1856 | Isolated | 2.59 |  |
| 9-hydroxyguatterfriesine | 340.1547 | Isolated | 2.28 |  |
| 7-hydroxyguatteriopsiscine | 358.1651 | Isolated | 2.32 |  |
| 9-hydroxyguattescine | 354.1702 | Isolated | 2.54 |  |
| 3-hydroxynornuciferine | 298.1437 | Isolated | 2.33 |  |
| 4-hydroxywilsonirine | 344.1493 | Isolated | 1.86 |  |
| imenine | 352.1181 | Isolated | 4.16 |  |
| isoboldine | 328.1551 | Isolated | 1.60 |  |
| isochondodendrine | 595.2810 | Isolated | 1.68 |  |
| isocorydine | 342.1703 | Isolated | 1.92 |  |
| isocorypalmine | 342.1701 | Isolated | 2.04 |  |
| isodaurisoline | 611.3116 | Isolated | 1.94 |  |
| isoguattouregidine | 342.1333 | Isolated | 2.10 |  |
| isolaureline | 309.13649 | Isolated | 2.72 |  |
| iso-9-methoxyguatterfriesine | 354.1697 | Isolated | 2.68 |  |
| isopiline | 298.1434 | Isolated | 2.29 |  |
| isopolyalthenol | 338.2477 | Isolated | 6.12 |  |
| isopycnarrhine | 192.1022 | Isolated | 0.82 |  |
| isotetrandrine | 623.3123 | Isolated | 2.21 |  |
| isotrilobine | 577.2698 | Isolated | 2.53 |  |
| jatrorrhizine | 338.1393 | Isolated | 2.47 |  |
| kikemanine | 342.1695 | Isolated | 1.77 |  |
| kohatine | 565.2322 | Isolated | 2.06 |  |
| krukovine | 595.2807 | Isolated | 1.74 |  |
| lanuginosine | 306.0761 | Isolated | 2.99 |  |
| laudanosine | 358.2015 | Isolated | 2.14 |  |
| laurifoline | 342.1705 | Isolated | 1.79 |  |
| laurolitsine | 314.1750 | Isolated | 1.53 |  |
| lauroscholtzine | 342.1696 | Isolated | 2.00 |  |
| laurotetanine | 328.1548 | Isolated | 2.07 |  |
| limacine | 609.2953 | Isolated | 2.10 |  |
| limacusine | 609.2958 | Isolated | 1.85 |  |
| limalongine | 334.1646 | Isolated | 0.97 |  |
| lindoldhamine | 569.2653 | Isolated | 1.77 |  |
| lirinidine | 282.1489 | Isolated | 2.22 |  |
| liriodenine | 276.0654 | Isolated | 2.66 |  |
| lycorine | 288.1226 | Isolated | 2.13 |  |
| lysicamine | 292.0973 | Isolated | 3.06 |  |
| macondine | 242.0809 | Isolated | 2.88 |  |
| magnocurarine | 314.1750 | Isolated | 1.37 |  |
| magnoflorine | 342.1701 | Isolated | 1.83 |  |
| menisperine | 356.1856 | Isolated | 1.96 |  |
| methoxyannomontine | 292.1197 | Isolated | 2.78 |  |
| methoxyatherosperminine | 340.1907 | Isolated | 3.46 |  |
| 3-methoxyanonaine | 296.1279 | Isolated | 2.79 |  |
| 10-methoxycaaverine | 298.1436 | Isolated | 2.29 |  |
| 3-methoxyguadiscine | 352.1544 | Isolated | 3.15 |  |
| 9-methoxyguatterfriesine | 354.1700 | Isolated | 2.61 |  |
| 3-methoxyguattescidine | 340.1176 | Isolated | 2.42 |  |
| 3-methoxyoxoputerine | 336.0867 | Isolated | 3.29 |  |
| 3-methoxyputerine | 326.1383 | Isolated | 2.68 |  |
| morphine | 286.1440 | Synthesis | 0.90 |  |
| N-Ac anonaine | 308.1284 | Isolated | 4.50 |  |
| nandigerine | 312.1228 | Isolated | 1.50 |  |
| nandinine | 326.1384 | Isolated | 2.34 |  |
| 2-norberbamine | 595.2798 | Isolated | 2.15 |  |
| 2’-norfuniferine | 609.2953 | Isolated | 1.95 |  |
| norlaureline | 296.1279 | Isolated | 2.44 |  |
| N-Ac nornantenine | 368.1490 | Isolated | 4.32 |  |
| N-formylduguevanine | 370.1286 | Isolated | 4.08 |  |
| N-formylputerine | 324.1232 | Isolated | 4.20 |  |
| N,N’-dimethyllindoldamine | 597.2963 | Isolated | 1.95 |  |
| N,N’-dimethylurabaine | 585.2744 | Isolated | 5.05 |  |
| neothalibrine | 625.3264 | Isolated | 2.25 |  |
| N-hydroxyannomontine | 278.1035 | Isolated | 3.07 |  |
| N-me actinodaphnine | 326.1381 | Isolated | 2.22 |  |
| N-me anomurine | 358.2015 | Isolated | 14.24 |  |
| N-me asimilobine | 282.1489 | Isolated | 2.30 |  |
| N-me coclaurine | 300.1592 | Isolated | 2.36 |  |
| N-me crotsparine | 298.1434 | Isolated | 1.19 |  |
| N-me dioncophylline A | 392.2216 | Isolated | 3.16 |  |
| N-me elmerrillicine | 326.1385 | Isolated | 2.02 |  |
| N-me isocorypalmine | 356.1855 | Isolated | 1.88 |  |
| N-me laurotetanine | 342.1703 | Isolated | 2.02 |  |
| N-me nandigerine | 326.1382 | Isolated | 1.76 |  |
| N-me pachypodanthine | 310.1439 | Isolated | 2.84 |  |
| N-me urabaine | 571.2585 | Isolated | 5.27 |  |
| norargemonine | 342.1704 | Isolated | 1.70 |  |
| noratherosperminine | 296.1647 | Isolated | 3.06 |  |
| norcycleanine | 609.2954 | Isolated | 2.23 |  |
| nordicentrine | 326.1387 | Isolated | 2.32 |  |
| norglaucine | 342.1700 | Isolated | 2.28 |  |
| 2’-norguattoguianine | 609.2960 | Isolated | 1.92 |  |
| norisocorydine | 328.1548 | Isolated | 1.81 |  |
| norisodomesticine | 312.1227 | Isolated | 2.02 |  |
| norjuziphine | 286.1434 | Isolated | 1.99 |  |
| 2’-norlimacusine | 595.2800 | Isolated | 2.02 |  |
| nornantenine | 326.1387 | Isolated | 2.50 |  |
| nornuciferidine | 298.1437 | Isolated | 2.48 |  |
| nornuciferine | 282.1488 | Isolated | 2.55 |  |
| noroliverine | 326.1388 | Isolated | 2.72 |  |
| norpurpureine | 372.1803 | Isolated | 2.55 |  |
| norstephanine | 296.1284 | Synthesis | 2.75 |  |
| northalicthuberine | 340.1544 | Isolated | 2.88 |  |
| northalifoline | 194.083 | Isolated | 1.14 |  |
| 2-northalmine | 595.2798 | Isolated | 1.99 |  |
| norushinsunine | 282.1127 | Isolated | 2.12 |  |
| noscapine | 414.1551 | Synthesis | 3.64 |  |
| nuciferine | 296.1645 | Isolated | 2.37 |  |
| O-acetylatheroline | 380.1131 | Isolated | 3.18 |  |
| 3-O-acetylvittatine | 314.138 | Isolated | 2.19 |  |
| obaberine | 623.3117 | Isolated | 2.35 |  |
| obamegine | 595.2803 | Isolated | 2.11 |  |
| obovanine | 282.112 | Isolated | 1.73 |  |
| oliveridine | 326.1394 | Isolated | 2.44 |  |
| oliveridine *N*-oxide | 342.1337 | Isolated | 2.66 |  |
| oliverine | 340.1546 | Isolated | 2.75 |  |
| oliverine *N*-oxide | 356.1496 | Isolated | 2.74 |  |
| oliveroline | 296.1288 | Isolated | 2.30 |  |
| O-methylarmepavine | 328.1911 | Isolated | 2.40 |  |
| O-methylatheroline | 352.1176 | Isolated | 2.87 |  |
| O-methylcocsoline | 563.2525 | Isolated | 2.49 |  |
| O-methylisopiline | 312.1595 | Isolated | 2.80 |  |
| O-methylmoschatoline | 322.1074 | Isolated | 4.20 |  |
| O-methylpukateine | 310.1442 | Isolated | 2.43 |  |
| oubacryptine | 350.1748 | Isolated | 2.35 |  |
| oxobuxifoline | 336.0867 | Isolated | 3.68 |  |
| oxoglaucine | 352.1181 | Isolated | 3.08 |  |
| oxonantenine | 336.0866 | Isolated | 3.14 |  |
| oxoputerine | 306.0762 | Isolated | 2.58 |  |
| oxosarcocapnidine | 324.0868 | Isolated | 3.27 |  |
| oxostephanine | 306.0767 | Isolated | 2.15 |  |
| oxyacanthine | 609.2959 | Isolated | 1.95 |  |
| oxypalmatine | 368.1493 | Isolated | 3.90 |  |
| pachyconfine | 298.1444 | Isolated | 1.66 |  |
| pachypodanthine | 296.1287 | Isolated | 2.43 |  |
| pachypodanthine N-acetyl | 338.1387 | Isolated | 4.60 |  |
| pachypodol | 345.0964 | Isolated | 4.67 |  |
| pachystaudine | 326.1389 | Isolated | 2.39 |  |
| pallidine | 328.1544 | Isolated | 1.34 |  |
| palmatine | 352.1550 | Isolated | 2.66 |  |
| pancoridine | 322.1071 | Isolated | 2.72 |  |
| pangkorimine | 565.2339 | Isolated | 1.52 |  |
| papaverine | 340.157 | Isolated | 2.31 |  |
| pessoine | 314.1382 | Isolated | 1.38 |  |
| phaeanthine | 623.3121 | Isolated | 2.49 |  |
| phyllocryptine | 342.1699 | Isolated | 2.16 |  |
| phyllocryptonine | 358.1646 | Isolated | 1.59 |  |
| polyalthenol | 338.2478 | Isolated | 6.74 |  |
| polycarpine | 386.1600 | Isolated | 3.91 |  |
| polyveoline | 340.2638 | Isolated3.91 | 3.67 |  |
| protopine | 354.136 | Isolated | 2.29 |  |
| pseudoanibacanine | 298.1440 | Isolated | 1.39 |  |
| pseudocolumbamine | 338.1392 | Isolated | 2.23 |  |
| pseudopalmatine | 352.1542 | Isolated | 2.63 |  |
| puterine | 296.1281 | Isolated | 2.39 |  |
| pycnamine | 609.2969 | Isolated | 2.07 |  |
| pycnarrhine | 192.1020 | Isolated | 0.78 |  |
| reticuline | 330.1703 | Isolated | 1.78 |  |
| reticuline Nα-oxide | 346.1651 | Isolated | 1.84 |  |
| reticuline N_β_-oxide | 346.1650 | Isolated | 1.84 |  |
| roemerialinone | 342.1696 | Isolated | 1.57 |  |
| roemerine | 280.1328 | Isolated | 2.58 |  |
| roemerolidine | 312.123 | Isolated | 2.06 |  |
| rurrebanidine | 314.1386 | Isolated | 2.04 |  |
| rurrebanine | 328.1544 | Isolated | 2.38 |  |
| sanguinarine | 332.0946 | Isolated | 4.13 |  |
| sarcocapnine | 342.1697 | Isolated | 2.32 |  |
| saxoguattine | 388.1754 | Isolated | 1.76 |  |
| scoulerine | 328.1541 | Isolated | 1.85 |  |
| sebiferine | 342.1703 | Isolated | 1.64 |  |
| sparsiflorine | 284.1281 | Isolated | 1.47 |  |
| splendidine | 322.1072 | Isolated | 4.29 |  |
| staudine | 532.2341 | Isolated | 3.20 |  |
| stephalagine | 310.1436 | Isolated | 2.86 |  |
| stephanine | 310.1438 | Isolated | 2.73 |  |
| stepharanine | 324.1232 | Isolated | 2.09 |  |
| stepharine | 298.1441 | Isolated | 1.58 |  |
| stepholidine | 328.1548 | Isolated | 1.56 |  |
| steponine | 342.1694 | Isolated | 1.75 |  |
| sternbergine | 332.142 | Isolated | 1.92 |  |
| stipitatine | 312.1594 | Isolated | 2.55 |  |
| stylopine | 324.1228 | Isolated | 2.56 |  |
| subsessiline | 338.1022 | Isolated | 3.53 |  |
| sukhodianine | 356.1486 | Isolated | 2.27 |  |
| takatonine | 354.1700 | Isolated | 3.62 |  |
| telobine | 563.2541 | Isolated | 2.40 |  |
| tembetarine | 344.1852 | Isolated | 1.58 |  |
| tetrahydropalmatine | 356.1865 | Isolated | 2.53 |  |
| tetrandrine | 623.3109 | Isolated | 2.24 |  |
| thalicthuberine | 354.1701 | Isolated | 2.96 |  |
| thalidasine | 653.3210 | Isolated | 2.51 |  |
| thalifaricine | 669.3157 | Isolated | 2.10 |  |
| thalifoline | 208.0968 | Isolated | 1.99 |  |
| thaligosine | 639.3054 | Isolated | 2.05 |  |
| thaliphylline | 609.2949 | Isolated | 2.06 |  |
| thaliporphine | 342.1704 | Isolated | 2.04 |  |
| thalrugosine | 609.2965 | Isolated | 2.16 |  |
| thebaine | 312.1594 | Isolated | 2.19 |  |
| tiliacorine | 577.2688 | Isolated | 2.15 |  |
| tiliacorinine | 577.2688 | Isolated | 2.21 |  |
| tricordatine | 549.2376 | Isolated | 1.85 |  |
| trigilletimine | 559.2229 | Isolated | 2.81 |  |
| trimethoxystyrene | 195.1015 | Isolated | 4.17 |  |
| triphyophylline | 378.2061 | Isolated | 3.28 |  |
| uvariopsine | 324.1593 | Isolated | 3.10 |  |
| 8-methoxyuvariopsine | 354.1700 | Isolated | 3.06 |  |
| velucryptine | 312.1226 | Isolated | 1.68 |  |
| vittatine | 272.1269 | Isolated | 2.02 |  |
| wilsonirine | 328.1547 | Isolated | 1.98 |  |
| xylopine | 296.1281 | Isolated | 2.17 |  |
| xylopinine | 356.1856 | Isolated | 1.91 |  |
| zenkerine | 298.1437 | Isolated | 2.21 |  |

# **Supplementary Table 1. Metabolites included in the IQAMDB collections**

Figure S1. Illustrative structures and scaffolds depicting rational clustering trends as discussed in the main text of the manuscript. The letters refer to the different clusters, as outlined in Figure 2.
